# Bayesian codon substitution modelling to identify sources of pathogen evolutionary rate variation

**DOI:** 10.1099/mgen.0.000057

**Published:** 2016-06-24

**Authors:** Guy Baele, Marc A. Suchard, Filip Bielejec, Philippe Lemey

**Affiliations:** ^1^​ Department of Microbiology and Immunology, Rega Institute, KU Leuven - University of Leuven, Leuven, Belgium; ^2^​ Departments of Biomathematics, Human Genetics and Biostatistics, David Geffen School of Medicine, University of California, Los Angeles, CA 90095, USA

**Keywords:** Codon substitution model, Hierarchical phylogenetic modeling, Intrahost HIV, Bat Rabies, Bayesian inference, Evolutionary rate variation

## Abstract

Phylodynamic reconstructions rely on a measurable molecular footprint of epidemic processes in pathogen genomes. Identifying the factors that govern the tempo and mode by which these processes leave a footprint in pathogen genomes represents an important goal towards understanding infectious disease evolution. Discriminating between synonymous and non-synonymous substitution rates is crucial for testing hypotheses about the sources of evolutionary rate variation. Here, we implement a codon substitution model in a Bayesian statistical framework to estimate absolute rates of synonymous and non-synonymous substitution in unknown evolutionary histories. To demonstrate how this model can provide critical insights into pathogen evolutionary dynamics, we adopt hierarchical phylogenetic modelling with fixed effects and apply it to two viral examples. Using within-host HIV-1 data from patients with different host genetic background and different disease progression rates, we show that viral populations undergo faster absolute synonymous substitution rates in patients with faster disease progression, probably reflecting faster replication rates. We also re-analyse rabies data from different bat species in the Americas to demonstrate that climate predicts absolute synonymous substitution rates, which can be attributed to climate-associated bat activity and viral transmission dynamics. In conclusion, our model to estimate absolute rates of synonymous and non-synonymous substitution can provide a powerful approach to investigate how host ecology can shape the tempo of pathogen evolution.

## Data Summary

1. Supplementary BEAST XML files have been deposited in figshare: 10.6084/m9.figshare.3385906

## Impact Statement

The tempo and mode of pathogen evolution can be shaped by a complex interplay of different factors impacting the generation and fixation rates of mutations in their genomes. Disentangling the neutral and selective contributions to evolutionary rate variation, and identifying their correlates, is a key challenge to gain insights into pathogen evolution and ecology. Here we develop a Bayesian evolutionary inference procedure to estimate absolute synonymous and non-synonymous substitution rates in rapidly evolving pathogens. By parameterizing these absolute codon substitution rates as a function of a set of potential covariates, the approach allows us to test which processes shape the tempo of pathogen evolution. We analyse within-host HIV-1 evolution in multiple patients and a multi-host bat rabies system to illustrate the power of our approach. We provide direct support for disease progression impacting absolute synonymous substitution rates in within-host HIV-1 evolution and for an association between host geography and absolute synonymous substitution rates in bat rabies lineages. Our implementation in a popular Bayesian software package allows for further refinements and extensions of the absolute synonymous and non-synonymous rates approach.

## Introduction

A complex interplay of processes that impact the generation and fixation of mutations in their genomes determines the tempo and mode of pathogen evolution. For rapidly evolving pathogens, including many RNA viruses, these processes can leave a molecular footprint on relatively short timescales. In fact, the central premise of phylodynamic inference is that the timescale of epidemic spread is commensurate with the evolutionary timescale of pathogens. This implies that rapidly evolving pathogen populations demonstrate measurable evolution and that time of sampling can be incorporated as a source of calibration in molecular clock models. This has proven extremely useful to reconstruct epidemic histories in calendar time units (Pybus & Rambaut, 2009), but identifying the factors that govern the speed of evolution is also of critical importance because it can lead to a better understanding of within-host evolution (Lemey *et al.*, 2007), transmission dynamics (Vrancken *et al.*, 2014) and viral emergence (Holmes, 2009).

Rates of evolution can vary dramatically among distantly related pathogen populations, such as different viral families. Both viral genomic features and ecological factors can explain such differences (Streicker *et al.*, 2012a; Hicks & Duffy, 2014). Mutation rates are determined by genome architecture and size (Belshaw *et al.*, 2008) and the ability to recombine may also contribute to high rates of evolution. Ecological factors such as target cell, transmission mode and host range, by contrast, can be responsible for heterogeneity in generation times and/or selective pressure. For example, vector-borne transmission of RNA viruses is associated with strong purifying selection in surface structural genes (Woelk & Holmes, 2002). More recently, Hicks & Duffy (2014) have shown that cell tropism, probably through its influence on virus generation time, predicts rates of mammalian RNA virus evolution. These examples indicate the importance of discriminating between synonymous and non-synonymous substitutions in explaining the source of evolutionary rate variation.

Among closely related viruses, variation in evolutionary rates may be more subtle, and hence more difficult to quantify accurately, as well as more challenging to explain because many of the factors mentioned above will be less variable at this scale. Nevertheless, recent studies demonstrate the interest in and the importance of assessing rate heterogeneity within closely related viral populations. Through the analysis of host-associated lineages of rabies virus in American bats, Streicker* et al.* (2012a) provide evidence for host ecology shaping the tempo of viral evolution, and at the within-host evolutionary level, HIV-1 substitution rates have been associated with disease progression (Lemey *et al.*, 2007; Edo-Matas* et al.*, 2011). Also in these cases, the explanations invoke different contributions of neutral evolution and selective dynamics. To quantify selective forces, much attention has been devoted to estimating the ratio of non-synonymous and synonymous rates. Indeed, natural selection operates most strongly at the protein level, implying that synonymous and non-synonymous mutations are fixed at very different rates due to substantial differences in selective pressure (Yang, 2006). When the focus also includes variation in the rate at which mutations are being generated, comparing changes in absolute synonymous and non-synonymous substitution rates can be more insightful. Although such an approach has been proposed for fixed topologies (Seo *et al.*, 2004), the computational burden associated with high-state space codon substitution models has hampered their development in popular Bayesian phylogenetic software accommodating phylogenetic uncertainty.

Sampling restrictions and stochastic error in the process of divergence accumulation may further complicate the difficulty in identifying more subtle evolutionary rate differences within viral populations. The range of sequence isolation times may be limited for many viruses, and the availability of samples generally decreases as we go back in time, which imposes a substantial departure from the ideal sampling design (Seo *et al.*, 2002). An overdispersed molecular clock represents an important source of stochastic error that will contribute to the uncertainty of independent evolutionary rate estimates for different viral lineages at various evolutionary scales. All these factors will considerably complicate formal testing of the association between evolutionary rate variation and covariate data. To maximize statistical efficiency, it may prove useful to integrate external covariate data into the phylogenetic inference procedure. This strategy has recently been pursued by Bayesian hierarchical modelling with fixed effects in the context of HIV-1 evolution in different patient groups (Edo-Matas *et al.*, 2011). Specifically, a Bayesian hierarchical phylogenetic model (HPM, Suchard *et al.*, 2003) was used to pool information across patients and improve estimate precision for patient-specific viral populations. By introducing fixed effects, evolutionary rate differences between specific populations could be formally assessed. In this case, patient assignment to specific patient groups representing different host genetic background or disease progression constituted the binary covariate data. A similar need for hypothesis testing emerged in the study on the evolutionary consequences of host switching in bat rabies viruses in the Americas (Streicker *et al.*, 2012a). While considerable variation in rabies evolutionary rates was noticeable among lineages associated with different hosts, accurate quantification remained difficult due to limited sequence samples and their variation across different host species. Using the same Bayesian modelling approach that integrates physiological, environmental and ecological traits as covariate data, Streicker *et al.* (2012a) were able to provide strong evidence for climate predicting rates of viral evolution, which is probably associated with seasonal activity of the bat host.

Here we focus on identifying covariates that may have a significant influence on different aspects of the evolutionary process. To this end, we adopt hierarchical phylogenetic modelling with fixed effects in a novel procedure to estimate absolute synonymous and non-synonymous substitution rates. We use the approach of Edo-Matas *et al.* (2011), which allows us to parameterize evolutionary rate parameters as log linear functions of various potential covariates and perform Bayesian model averaging over all possible combinations of covariates. We demonstrate how we can more directly test hypotheses about evolutionary rate variation using examples of within-host HIV-1 evolution and bat rabies dynamics.

## Methods

Our Bayesian evolutionary inference approach is based on a standard codon substitution model (MG94, Muse & Gaut, 1994) with rate variation among sites modelled using a discretized gamma distribution (Yang, 1996). The MG94 codon model posits that substitutions between nucleotide triplets along a branch of the evolutionary history occur according to a continuous-time Markov chain (CTMC) process with infinitesimal rate matrix $Q=\left\{q_{ij}\right\}$ with non-negative off-diagonal elements $q_{ij}$ for i,j=1,.......,N where is the number of non-stop codons in the genetic code. We parameterize $q_{ij}$ following Hasegawa *et al.* (1985) such that κ is the nucleotide transition/transversion rate ratio and introduce the absolute synonymous substitution rate α and the absolute non-synonymous substitution rate *β*. Specifically, we set:

$$q_{ij}=\left\{ \begin{array}{rl} \frac{\alpha }{c_{S}}\kappa \Pi_{n}, & i\to j\;is\;a\;one\;-\;nucleotide\;synonymous\\ & \;\;transition\;to\;codon\;\Pi_{n}\\ \frac{\alpha }{c_{S}}\Pi_{n}, & i\to j\;is\;a\;one\;-\;nucleotide\;synonymous\\ & \;transversion\;to\;codon\;\Pi_{n}\\ \frac{\beta }{C_{N}}\kappa \Pi_{n}, & i\to j\;is\;a\;one\;-\;nucleotide\;non\;-\;synonymous\\ & \;\;transition\;to\;codon\;\Pi_{n}\\ \frac{\beta }{C_{N}}\Pi_{n}, & i\to j\;is\;a\;one\;-\;nucleotide\;non\;-\;synonymous\\ & \;transversion\;to\;codon\;\Pi_{n}\\ 0, & multiple\;substitutionsneeded \end{array}\right.$$

where $c_{s}$ is the number of possible synonymous changes and $c_{N}$ is the number of possible non-synonymous changes in the genetic code and $\Pi_{n}$ is the empirical frequency in the data of codon n=1,...,N. Note that $\alpha /c_{S}$ and $\beta /c_{N}$ are per-codon rates. Typical nucleotide and codon substitution models are normalized so that one change per base or codon is expected in one unit of time (Felsenstein, 2004). We here apply a different standardization that corresponds with α+β expected rate changes per time unit. The program beast (Drummond *et al.*, 2012) allows estimating time-measured trees and we hence express the total substitution rates of our MG94 model in units of time^−1^ used to specify the sampling dates of the data sequences.

We employ an HPM with fixed effects (Edo-Matas *et al.*, 2011) on each absolute rate of synonymous and non- synonymous substitution and hence parameterize each of those absolute rates as a log linear function of their potential predictors. All continuously valued predictors are log-transformed and standardized prior to specifying them as potential covariates. For each predictor k=1,...,K, with* K* the total number of potential predictors, our HPM parameterization with fixed effects includes a coefficient $\theta_{k}$, which quantifies the contribution or effect size of the predictor (in log space), and a binary indicator variable $\delta_{k}$, which allows the predictor to be included or excluded from the model. We estimate the inclusion variables using a Bayesian stochastic search variable selection (BSSVS) (Kuo & Mallick, 1998), resulting in an estimate of the posterior inclusion probability or support for each predictor. We assume a multivariate normal prior, centred at zero, on the coefficients $\theta_{k}$, with a diagonal precision matrix with all entries equal to 2. We also assume a vague (or diffuse) normal prior, centred at zero, on the intercept of the HPM, with precision equal to 10^−3^. We consider vague (or diffuse) priors as prior distributions that are not very informative and therefore have a minimal effect on posterior estimates. We assign independent Bernoulli prior probability distributions on $\delta_{k}$ and use a small prior probability on each predictor’s inclusion that reflects a 50 % prior probability on no predictors being included (Lemey *et al.*, 2014).

Analogous to Edo-Matas *et al.* (2011) and Lemey *et al.* (2014), we can use Bayes factors (BFs) (Kass & Raftery, 1995) to express how much the data change our prior opinion about the inclusion of each predictor. These BFs are calculated by dividing the posterior odds for the inclusion of a predictor with the corresponding prior odds. Kass & Raftery (1995) provide guidelines for assessing the strength of the evidence against our prior opinion: BFs between 1 and 3 are not worth more than a bare mention, while values between 3 and 20 are considered positive evidence against our prior opinion. BFs in the ranges 20–150 and >150 are considered to be strong and very strong evidence against our prior opinion, respectively. In our results, we also report the mean and 95 % highest posterior density interval (HPD) for the conditional effect size for each predictor, which is the effect size conditional on the predictor being included in the model ($\theta_{k}/\delta_{k}=1$) .

In the analyses we perform in this paper, α and β are stratum-specific (e.g. patient-specific, lineage-specific in a tree or any other independent realization of the evolutionary process) parameters, whereas $\Pi_{n}$ and k are global parameters shared among all strata. We assume a stratum-specific distribution to model site rate heterogeneity and use γ to denote the shape parameter of the underlying gamma distribution. We adopt hierarchical modelling (Suchard *et al.*, 2003), which enables pooling information across data partitions to improve estimate precision in individual partitions, on these stratum-specific γ parameters that describe the rate variation among sites. We assume an underlying lognormal distribution, with a vague normal prior centred at zero on its mean and a vague gamma prior on its precision. We assume independent constant population size models for each stratum.

For our bat rabies data set (see the Data section below), we also explore the use of a stratum-specific two-epoch constant–constant coalescent model (Edwards *et al.*, 2006) and specify hierarchical priors on both constant population size parameters. We consider the difference between the time to the most recent common ancestor (TMRCA) and the transition time, which is the time between the two epochs in the coalescent model, as the ‘lag time’, which we use as a predictor in the generalized linear model parameterization. Given that using this lag time in turn as a predictor in the HPM for our absolute synonymous and non-synonymous rates can lead to identifiability issues, we employ an empirical tree distribution and include a proposal mechanism in our Markov chain Monte Carlo (MCMC) analysis that randomly draws a new tree from this empirical distribution (Pagel *et al.*, 2004; Lemey* et al.*, 2014).

Posterior distributions were obtained using Bayesian inference through MCMC as implemented in beast v1.8.3 (Drummond *et al.*, 2012). MCMC chains were run for sufficiently long to ensure stationarity and adequate effective sample sizes > 100 as diagnosed using Tracer 1.6 (http://tree.bio.ed.ac.uk/software/tracer/). Phylogenetic likelihood computations for large state-space models such as codon substitution models impose considerable computational burden. We effectively deal with this by evaluating the phylogenies on graphics processing units (GPUs) through the use of beagle (Ayres *et al.*, 2012) in combination with beast, making use of the large number of processing cores on GPUs to efficiently parallelize calculations (Suchard & Rambaut, 2009).

As a first data set, we revisit a within-host HIV-1 data set containing nucleotide sequences for the gp120 (C1–C4) from virus populations in 18 men who have sex with men (MSM) participants, 11 with a WT genotype and seven with a CCR5 wt/Δ32 genotype. Nine individuals were classified as long-term non-progressors (LTNP) and the remaining nine individuals progressed to AIDS during the study period and were classified as progressors (P). For more details on this data set, readers are referred to Edo-Matas *et al.* (2011). We use the following predictors in the HPM with fixed effects for both the absolute synonymous and non-synonymous substitution rates: progressor status (P/LTNP), genotype (WT/CCR5 wt/Δ32 genotype), and sampling characteristics including the log of the number of sequences and log of the sampling time intervals.

As a second data set, we analyze the bat rabies data set of Streicker *et al.* (2012a), containing 648 sequences from the coding region of the N gene, collected between 1972 and 2009 from 21 bat species or sub-species. Streicker *et al.* (2012a) collected information on the overwintering activity patterns, migratory behavior, roosting behavior and metabolic rates – basal (BMR) and during seasonal torpor (TMR) – of the bat species that served as reservoir hosts for the rabies viruses included here from the primary literature and existing databases. We use the following predictors in the HPM with fixed effects on the absolute synonymous and non-synonymous substitution rates: climate region (temperate/subtropical), log of the mass-independent BMR, log of the mass-independent TMR, colonial aggregation (solitary/colonial), winter activity pattern (hibernation/aseasonal), long distance migration (none/long distance), and sampling characteristics including the log of the number of sequences and log of the sampling time intervals.

## Results

### Intrahost HIV-1 evolution

**Fig. 1.**
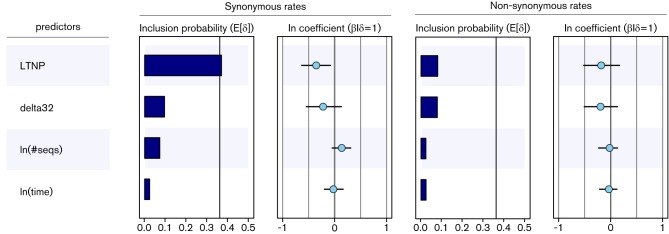
Predictors of the absolute synonymous and non-synonymous substitution rates of the MG94 codon model for the within-host HIV-1 data set. The inclusion probabilities are defined by the indicator expectations E[δ] because they reflect the frequency at which the predictor is included in the model and therefore represent the support for the predictor. An indicator expectation corresponding to a BF support value of 3 is represented by a thin vertical line. The contribution of each predictor, when included in the model (β/δ=1), where β is the coefficient or conditional effect size, is represented by the mean and credible intervals of the HPM coefficients on a log scale.

We evaluate the contribution of several potential predictors to the variation in absolute synonymous and non-synonymous substitution rates among patients (Fig. 1). Our analysis reveals that only disease progression status is associated with absolute synonymous substitution rate under the MG94 model. We find positive evidence that disease progression status contributes to the synonymous substitution process of our within-host HIV-1 data set. This is reflected in the conditional effect size of this covariate [−0.35 on a log scale; accompanying 95 % HPD: (-0.64; −0.07)] and the statistical support for its inclusion in the model (posterior probability = 0.37 and BF = 3). CCR5 genotype of the host, the number of sequences and the time interval of sampling do not yield appreciable support. None of the predictors yield appreciable support for an association with absolute non-synonymous substitution rates. We explicitly included the numbers of sequences and the number of time points per patient to test whether sampling bias may impact the estimates.

### Multi-host bat rabies evolution

**Fig. 2.**
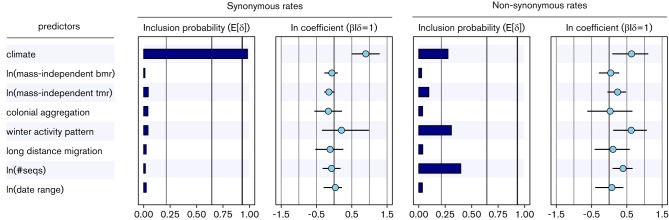
Predictors of the absolute synonymous and non-synonymous substitution rates of the MG94 codon model for the bat rabies data set. The inclusion probabilities are defined by the indicator expectations E[δ] because they reflect the frequency at which the predictor is included in the model and therefore represent the support for the predictor. Indicator expectations corresponding to BF support values of 3, 20 and 150 are represented by thin, medium thick and thick vertical lines, respectively. The contribution of each predictor, when included in the model (β/δ=1), where β is the coefficient or conditional effect size, is represented by the mean and credible intervals of the HPM coefficients on a log scale.

We evaluate the contribution of physiological, environmental and ecological predictors on absolute synonymous and non-synonymous substitution rate variation among host-associated bat virus lineages (Fig. 2). Our analysis reveals that only the environmental climatic trait contributes to absolute synonymous substitution rate variation among the bat rabies lineages. This is reflected in the conditional effect size of this covariate (0.90 [0.50; 1.29], reflecting a 2.5-fold increase of the synonymous substitution rate for those bats in subtropical regions as compared with those in temperate regions) and the very strong statistical support for its inclusion in the model (posterior probability = 0.98 and BF = 591). None of the other predictors yields appreciable support for an association with absolute synonymous substitution rates. We also find some support for a number of predictors on absolute non-synonymous rate variation. Climate region, winter activity pattern and the number of sequences yield a BF of 4, 5 and 7, respectively, and they all result in positive conditional effect sizes (0.69 [0.16; 1.21], 0.61 [0.05; 1.14] and 0.39 [0.15; 0.66], respectively).

The association between climatic region of the host and viral absolute synonymous substitution rates agrees with the finding of Streicker *et al.* (2012a) of slower molecular clock ticking at the third codon position of rabies viral gene sequences sampled from temperate bat species as compared with those sampled from tropical and subtropical host species. The authors also argue against a metabolism-mediated relationship between environmental temperature and viral replication, but claim that generation times between viral infections probably differed among climatic regions. Specifically, year-round transmission and replication may increase the annual number of viral generations in tropical and subtropical bats relative to seasonal pulses of transmission in temperate species, thereby accelerating evolution in these hosts. In this respect, it may be surprising that winter activity pattern, as a more direct measure of reduced seasonality, is not included in the model as a covariate of absolute synonymous rate variation. However, Streicker *et al.* (2012a) argue that seasonal activity is poorly understood for many bat species (Dunbar & Brigham, 2010), and overwintering records frequently have to generalize from a few observations to an entire species range, which can span wide areas. Climate may therefore serve as a better proxy for seasonality than current overwintering records.

**Fig. 3.**
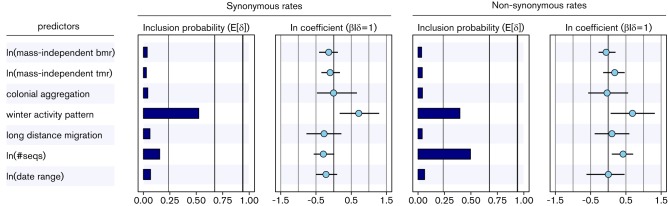
Predictors of the absolute synonymous and non-synonymous substitution rates of the MG94 codon model for the bat rabies data set. The inclusion probabilities are defined by the indicator expectations E[δ] because they reflect the frequency at which the predictor is included in the model and therefore represent the support for the predictor. Indicator expectations corresponding to BF support values of 3, 20 and 150 are represented by thin, medium thick and thick vertical lines, respectively. The contribution of each predictor, when included in the model (β/δ=1), where β is the coefficient or conditional effect size, is represented by the mean and credible intervals of the HPM coefficients on a log scale.

To investigate this in more detail, we perform the same analysis without the climate region predictor (Fig. 3). This analysis reveals that winter activity pattern is now associated with both synonymous and non-synonymous absolute substitution rates. We estimate a conditional effect size for the winter activity predictor of 0.71 [0.17; 1.29] and 0.69 [0.07; 1.32], respectively, and a BF support for its inclusion of 11 and 6, respectively. The fact that the winter activity predictor is included in the model in the absence of climate, but with much lower support and somewhat lower conditional effect size than climate, is in line with the argument that climate is a better proxy of seasonal activity.

**Fig. 4.**
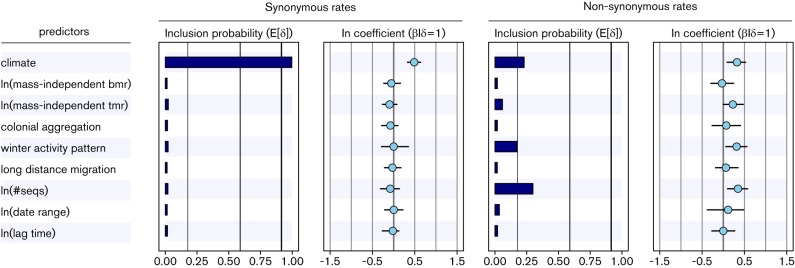
Predictors of the absolute synonymous and non-synonymous substitution rates of the MG94 codon model for the bat rabies data set. The inclusion probabilities are defined by the indicator expectations E[δ] because they reflect the frequency at which the predictor is included in the model and therefore represent the support for the predictor. Indicator expectations corresponding to BF support values of 3, 20 and 150 are represented by thin, medium thick and thick vertical lines, respectively. The contribution of each predictor, when included in the model (β/δ=1), where β is the coefficient or conditional effect size, is represented by the mean and credible intervals of the HPM coefficients on a log scale.

Finally, we also consider an additional predictor for absolute substitution rates motivated by a study on the adaptive dynamics underlying bat virus host shifts giving rise to host-associated viral lineages (Streicker *et al.*, 2012b). Their study showed that lineages involving greater numbers of positively selected substitutions have longer delays between cross-species transmission and enzootic viral establishment. This delay or lag time is estimated using a two-epoch coalescent model (Edwards *et al.*, 2006). Here, we incorporate this coalescent model in our Bayesian framework and include the lag time estimate as a potential covariate for the substitution rates. Due to the specific setup of this model (see Methods), we present this as a separate analysis. The analysis does not yield any evidence for a contribution of lag time to the absolute rates of synonymous and non-synonymous substitution in our MG94 model (Fig. 4), and produces results that are otherwise highly similar to those without the additional predictor (Fig. 2).

## Discussion

Here we present a novel implementation of the MG94 codon model (Muse & Gaut, 1994) in beast, allowing us to estimate absolute synonymous and non-synonymous substitution rates and parameterize each of those absolute rates as a log linear function of their potential predictors. This MG94 parameterization leads to separate absolute rate estimates whereas the alternative GY94 codon model (Goldman & Yang, 1994) only allows estimating the non-synonymous/synonymous rate ratio. To be able to estimate absolute rates, our adaptation of the MG94 model deviates from the standard assumption of one expected change per base per unit of time. Because of its availability in beast (Drummond *et al.*, 2012), our MG94 codon model can take full advantage of the computationally efficient codon implementation in the beagle library (Ayres *et al.*, 2012) to speed up the likelihood calculations.

In this paper, we show that our model can be combined with the HPM with fixed effects approach of Edo-Matas *et al.* (2011) to test specific evolutionary hypotheses. Our approach of mixed effects modelling on absolute synonymous and non-synonymous substitution rates offers a more direct and refined way of testing hypotheses concerning the tempo and mode of evolution as compared with the earlier uses of mixed effects modelling (Edo-Matas *et al.*, 2011; Streicker *et al.*, 2012a). Edo-Matas *et al.* (2011) used an HPM with fixed effects to test for differences in the overall rate of viral evolution between HIV-1-infected patients with different CCR5 host genetic background and disease progression. The authors found a weak association between evolutionary rate and disease progression, but to argue that this difference was due to differences in viral replication rates and hence generation time, they had to perform an additional analysis to show that these differences could not be attributed to selective dynamics. The results we obtained by directly and independently modelling these patient group effects on the absolute synonymous and non-synonymous substitution rates under our MG94 model adaptation corroborate the findings of Edo-Matas *et al.* (2011). We note that differences in generation time due to differences in replication rates are also expected to impact absolute non-synonymous substitution rates. Selection, however, may act as a confounding factor to detect such differences, in particular in the gene region targeted by neutralizing antibodies that we analyse here. Together with the fact that the statistical support was only moderate for absolute synonymous rates, it is therefore not surprising that we did not find any impact of disease progression on non-synonymous absolute substitution rate variation.

In their multi-host bat rabies analysis, Streicker *et al.* (2012a) used an HPM with fixed effects on the evolutionary rate parameters at the third codon position, as a proxy for synonymous evolution. Our method avoids the use of such proxies and makes use of the full data. Using the covariate modelling approach of Edo-Matas *et al.* (2011), Streicker *et al.* (2012a) modelled evolutionary rate parameters for 21 bat rabies virus lineages as a function of the aforementioned predictors, which allowed them to simultaneously estimate (conditional) effect size and posterior inclusion probability (and hence BF support) for the individual predictors. The authors found strong support for accelerated viral evolution in the tropics and subtropics relative to viruses restricted to the temperate zone, with negligible support for all other predictors. By modelling covariate effects directly and independently on the absolute rates of synonymous and non-synonymous substitutions in a codon substitution model, we corroborate the findings of Streicker *et al.* (2012a) and reinforce their interpretation of the rate difference. In this case, the viral generation time effect of reduced seasonality also has an impact on the absolute non-synonymous rates, albeit less strongly so. In agreement with the argument that climate may be a more accurate descriptor for seasonality than current overwintering records, we also found positive support for winter activity pattern on both absolute rates when climatic region was removed as a potential predictor, but with weaker support (BF = 11 vs. BF = 591). Climatic region may be particularly more appropriate as a proxy for seasonality for species that demonstrate geographically variable overwintering behaviours (McNab, 1974).

In addition to the original covariates, we also include sampling characteristics as potential predictors of the absolute substitution rates in our analyses as it may be argued that the more sequences sampled, and especially the longer the timespan over which they are sampled, the better the temporal signal will be, and the more accurately the substitution rate will be estimated (Seo *et al.*, 2002). For example, if lower temporal signal would lead to rate underestimation, sampling could bias our hypothesis testing for both absolute synonymous and non-synomous rates. This does not appear to be the case in our analyses, but the number of sequences is to some extent positively correlated with absolute non-synonymous substitution rates in the bat rabies analysis. This may reflect the impact of deleterious mutational load on the tip branches, which comprises a substantial proportion of the amino acid variation observed in natural populations of RNA viruses (Pybus *et al.*, 2007). In a separate rabies analysis, we also tested whether lag time, or the delay between cross-species transmission and enzootic viral establishment, as measured based on the transition time in a two-epoch coalescent model (Edwards *et al.*, 2006), had an effect on the absolute non-synonymous rates. Longer lag times were previously associated with a higher number of positively selected sites (Streicker *et al.*, 2012b). It may not be surprising that we did not detect an effect of lag times in our analyses as the number of positively detected sites involved in host species adaptation was found to be limited to six codons in the coding N gene region we analyse [of which there were indications that these were false positives (Streicker *et al.*, 2012b)] and hence unlikely to impact overall non-synonymous substitution rates. In addition, our analysis is based only on the coding N gene region that was studied in the original multi-host rabies evolutionary rate analysis (Streicker *et al.*, 2012a), whereas the selection analysis was based on the N, G and L genes, leaving us with less data to capture the signal of adaptation (Streicker *et al.*, 2012b).

Our implementation of the MG94 model with absolute rates paves the way for further codon model extensions in a Bayesian framework. An important avenue of future research would be to allow for site-specific and lineage-specific variation of both the absolute synonymous and non-synonymous substitution rates (e.g. Pond & Muse, 2005). Not only would this allow us to detect positive selection at specific sites or branches, but it would also more appropriately model long-term purifying selective pressure and result in more accurate deep divergence dating for viruses (Wertheim & Pond, 2011). The additional model complexity may, however, be associated with substantial computational burden, but massively parallel likelihood computations on multi-core architecture using an efficient framework such as beagle (Ayres *et al.*, 2012) may offer a solution to this.
